# Functional and structural characterization of the SARS-CoV-2 spike N481K mutation

**DOI:** 10.1007/s00705-026-06652-y

**Published:** 2026-06-04

**Authors:** Maria K. Smatti, Hebah Al-Khatib, Muhammad Suleman, Muna Nizar, Hala Abo Khalid, Ruaa Ismail, Gheyath K. Nasrallah, Hadi M. Yassine

**Affiliations:** 1 Biomedical Research Center, QU Health, Qatar University, PO Box 2713, Doha, Qatar; 2 Department of Biomedical Science, College of Health Sciences, QU Health, Qatar University, PO Box 2713, Doha, Qatar; 3 Laboratory of Animal Research Center (LARC), Qatar University, PO Box 2713, Doha, Qatar; 4 WHO Collaborating Center for Research & Capacity Building on Emerging & Re-emerging Zoonotic Diseases, Qatar University, PO Box 2713, Doha, Qatar

## Abstract

Background: Selective pressure drives SARS-CoV-2 evolution, with spike RBD substitutions shaping ACE2 binding and antibody escape. N481K RBD mutation emerged early in Qatar (2020) and later re-emerged globally, reaching near-fixation by 2024–2025, yet its functional significance remained unexplored. Methods: We analyzed the temporal and geographic circulation of N481K across > 17 million SARS-CoV-2 genomes (2020–2025). Structural effects were assessed using modeling, docking, and molecular dynamics. Functional impact was evaluated using VSV pseudovirus neutralization tests with sera from infected individuals (n = 48) and vaccinated cohorts: mRNA (n = 16), Sinopharm (n = 45), and AstraZeneca (n = 28). Results: N481K was detected in 4.14% (703,777/17,016,111) of global genomes and reached near-fixation (> 90%) in dominant Omicron lineages by 2024, with prevalence rising from 71.5% in early 2024 to 94.4% by week 36 of 2025. Structural analyses showed a 0.706 Å RMSD deviation and higher ACE2 docking affinity (–313 vs. − 308 kcal/mol) compared to the wild-type (WT) RBD. Molecular dynamics confirmed increased stability and lower binding free energy for N481K versus WT (–77.07 ± 0.90 vs. − 43.09 ± 0.98 kcal/mol). Among samples with detectable WT neutralization (n = 89), mean neutralization declined from 69.76% (WT) to 42.47% (N481K) (p < 0.0001), with the largest reductions in AstraZeneca, Sinopharm, and infected cohorts. mRNA recipients retained the highest neutralization [77.5% (WT) vs. 56.0% (N481K)]. Conclusion: N481K enhances ACE2 binding while partially evading antibody neutralization, suggesting viral fitness and persistence.

**Supplementary Information:**

The online version contains supplementary material available at 10.1007/s00705-026-06652-y.

## Introduction

Severe acute respiratory syndrome coronavirus 2 (SARS-CoV-2), responsible for the global COVID-19 pandemic, with over 771 million confirmed cases and more than 7 million deaths as of September 2025, according to the data of the World Health Organization (WHO). More than four years into the COVID-19 pandemic, SARS-CoV-2 continues to evolve through the accumulation of mutations that shape virus transmission, host adaptation, and immune escape. Specifically, the viral spike (S) protein, which mediates the interaction between the virus and host cells, undergoes numerous amino acid substitutions driven by intense immune pressure resulting from natural infection, vaccination, or antibody-based treatments [1–3]. Additionally, substitutions in the receptor-binding domain (RBD), corresponding to amino acid residues 306–534 of the S protein, have been the most consequential, as they directly modulate the interaction with ACE2 receptor or antibodies [4].

Deep-mutational scanning confirms that many escape mutations in the RBD impose a trade-off between antibody evasion and receptor binding, while others fix beneficial combinations that restore or strengthen affinity [5]. Not only that, structural and functional analyses highlight that non-RBD spike mutations can modulate RBD conformation and thus indirectly influence both ACE2 binding and antibody sensitivity [5]. In the RBD, mutations such as K417N/T, E484K, L452R, T478K, and N501Y have been shown to alter spike–ACE2 binding dynamics and antibody recognition, enabling the virus to adapt to immune pressure while maintaining efficient receptor engagement [3, 6–10]. These evolutionary adaptations have been central to the emergence and global dominance of major variants of concern (VOCs), including Alpha, Beta, Delta, and Omicron lineages [5, 11].

While extensive work has focused on canonical RBD mutations, other substitutions remain poorly characterized despite their increasing prevalence in globally circulating variants. One such mutation is the N481K, which is located within the receptor-binding motif (RBM), the most exposed and immunologically sensitive region of the RBD [12]. The substitution introduces a positively charged lysine residue that could influence the local electrostatic environment of the RBD–ACE2 interface and potentially affect antibody accessibility. This mutation has been sporadically detected since the early 2020, with the highest prevalence in Qatar, as reported in ~ 17% of B.1.428 and ~ 8% of B.1 genomes [13]. Although this mutation initially circulated locally before disappearing, it later reemerged and became increasingly prevalent in Omicron-descendant lineages globally (CoV-Spectrum, 2025). Despite its high circulation, the structural and functional consequences of N481K have not been fully investigated. To address this gap, the present study investigates the circulation, structural consequences, and neutralization phenotype of the N481K mutation in infected and vaccinated individuals.

## Methodology

### Genomic surveillance of S: N481K mutation

Publicly available SARS-CoV-2 genomic data were analyzed using the CoV-Spectrum platform (https://cov-spectrum.org). Sequences deposited between January 6, 2020 and September 10, 2025, were included. The N481K substitution in the spike protein was queried using the following parameters: S:N481K mutation filter, all lineages, global dataset, and sampling dates from January 6, 2020 to September 10, 2025. The outputs from CoV-Spectrum were used to construct temporal prevalence plots and geographic distribution figures that illustrate the emergence of N481K in 2020, its subsequent disappearance, and its re-emergence from 2023 onward.

### Molecular docking and structural modeling of S: N481K mutation

#### Mutants modeling and superimposition

To explore the impact of the N481K mutation on the interaction between the spike protein and the human ACE2 receptor, we retrieved the sequence of the RBD spanning amino acids 319 to 541 from the wild-type spike protein (P0DTC2), available in the UniProt database [14]. Using the Chimera software with modeler 14.0, we generated the 3D structure of the RBD [15, 16]. Employing an in-silico mutagenesis approach, we simulated structural changes to comprehend their potential impact on binding affinity and disease mechanisms [6, 10]. To assess how the mentioned mutation affects the structure and functionality of RBD proteins, we utilized the Chimera software to incorporate the N481K mutation into the wild-type structures. To conduct a comparative analysis between the wild-type and mutant RBD, we aligned the mutant proteins with the wild-type structure and quantified the variations using Root Mean Square Deviation (RMSD).

#### Molecular docking of wild-type and mutant RBD and ACE2

To investigate the impact of the identified mutation on the bonding network between the wild-type and N481K RBD with the human ACE2 receptor we utilized the HDOCK server (http://hdock.phys.hust.edu.cn/) [17]. Unlike other ab-initio docking approaches, HDOCK stands out by employing a hybrid algorithm that combines template-based modeling and ab initio free docking. This versatile server is capable of conducting docking for various molecular interactions, such as protein-protein and protein-ligand interactions. In this particular study, the docking between the RBD and ACE2 was executed by specifying the interaction residues, including 449:A, 453:A, 455:A, 456:A, 486:A, 487:A, 489:A, 493:A, 496:A, 498:A, 500:A, 501:A, 502:A, 505:A for RBD, and 21:B, 24:B, 27:B, 28:B, 30:B, 35:B, 38:B, 79:B, 80:B, 82:B, 83:B, 353:B for ACE2 [18]. Subsequently, the complexes generated by HDOCK were analyzed using the PDBsum web server (http://www.ebi.ac.uk/thornton-srv/databases/pdbsum/Generate.html) [19] to visualize the bonding interface, encompassing hydrogen bonds, salt bridges, and non-bonded contacts.

#### Molecular dynamics of wild-type and N481K complexes

To assess the structural conformations of the generated complexes within a dynamic environment, we employed the amber20 package along with the FF19SB force field [20, 21] to conduct molecular dynamics simulations of wild-type and N481K complexes. These complexes were placed within a Tip3 water box (10Å) and neutralized by the addition of Na + and Cl- ions. To address clashes within the system, a two-step gentle energy minimization procedure was implemented. Initially, energy minimization was carried out using the steepest descent and conjugate gradient algorithms for 12,000 and 6000 cycles, respectively. Subsequently, a second energy minimization step involved 6000 and 3000 cycles, respectively [22, 23]. After performing energy minimization, the system underwent equilibration and was heated under constant pressure (1 atm) at 300 K. A subsequent 200ns molecular dynamics simulation employed the particle mesh Ewald algorithm to handle long-range electrostatic interactions and the SHAKE algorithm to manage covalent interactions. Post-simulation analysis was then conducted on the trajectories generated during the molecular dynamics simulation [10, 24].

#### Post-simulation analysis

CPPTRAJ (C++ Program for Trajectory Analysis) and PTRAJ (Program for Trajectory Analysis) packages were employed to assess how the N481K variant impacts the compactness, flexibility, hydrogen bonds, and dynamic stability between the wild-type and mutant complexes [25]. To examine structural compactness during the simulation period, the radius of gyration (Rg) was computed. Additionally, we calculated the RMSD to analyze structural dynamic stability. This calculation utilized the following formula:

1 $$RMSD\;=\;\sqrt{\frac{\sum_{i=0}^N\left[m_i\;\ast\left(X_i\;-\;Y_i\right)^2\right]}M}$$

Here, N represents the number of atoms, m_i_ represents the mass of atom i, X_i_ denotes the coordinate vector for the target atom i, Y_i_ represents the coordinate vector for the reference atom i, and M is the total mass. For assessing structural flexibility at the residue level, Root Mean Square Fluctuation (RMSF) was computed. Unlike RMSD, which examines positional differences across the entire complex, RMSF calculates residual fluctuation during the simulation period.

#### Binding free energy calculation

We employed the MM/PBSA (Molecular Mechanics / Poisson–Boltzmann Surface Area) approach to dynamically calculate real-time binding free energies for both wild-type and mutant RBD-ACE2 complexes [26]. This methodology, deemed optimal for assessing binding free energies in a variety of biological complexes, including protein-protein, protein-nucleic acid, and protein-ligand interactions, was implemented using the MMGBSA.py script. The script calculated binding free energy by considering electrostatic, van der Waals, solvent-accessible surface area (SA), and generalized Born (GB) contributions for both the wild-type and mutant complexes. The corresponding mathematical equation employed for this computation is as follows:

2 $$\triangle G\;\left(bind\right)\;=\;\triangle G\left(complex\right)\;-\;\left[\triangle G\left(receptor\right)\;+\;\triangle G\left(ligand\right)\right]$$

However, to calculate each component separately, the following equation was used:

3 $$G\;=\;Gbond\;+\;Gele\;+\;GvdW\;+\;Gpol\;+Gnpol$$

### Functional characterization of the S: N481K mutation

#### Sample collection

A total of 137 samples were collected from individuals who had either been infected with SARS-CoV-2 (*n* = 48) or fully vaccinated against COVID-19. The vaccinated group included recipients of mRNA vaccines, Pfizer (BNT162b2, *n* = 11) and/or Moderna (mRNA-1273, *n* = 5), as well as those who received the adenoviral vector vaccine AstraZeneca (ChAdOx1-nCoV-19, *n* = 28) and the inactivated virus vaccine Sinopharm (BBIBP-CorV, *n* = 45). Samples from infected individuals were collected 2 weeks after the onset of symptoms. For vaccinated groups, samples from Sinopharm recipients were collected at least 14 days post-vaccination (range 2 weeks − 6 months). AstraZeneca samples were obtained at least 3 weeks post–second dose (range 21–91 days). Similarly, samples from mRNA-vaccinated individuals were collected at least two weeks post vaccination with the second dose.

#### Plasmid construction

The N481K (c1443g) mutation was introduced into the SARS-CoV-2 spike expression plasmid (Wuhan-Hu-1 spike), which was kindly provided by the Viral Pathogenesis Laboratory, Vaccine Research Center, National Institute of Health (NIH). As previously described, site-directed mutagenesis using the QuickChange Lightning Site-Directed Mutagenesis Kit (Agilent Technologies) was used according to the manufacturer’s instructions [27, 28]. Briefly, mutant strands were synthesized by thermal cycling with mutagenic primers containing the desired substitution. The parental DNA template was digested with Dpn I, and the resulting plasmid was transformed into *E. coli*. Successful incorporation of the mutation was verified by Oxford Nanopore Technologies (ONT) sequencing using the ligation sequencing kit (SQK-LSK109) prior to large-scale plasmid amplification. Plasmid DNA was subsequently purified using the PureLink™ HiPure Plasmid DNA Purification Kit (Thermo Fisher Scientific). Final DNA preparations were stored at − 20 °C until use.

#### Generation of VSV pseudotypes and neutralization assays

The recombinant ΔG Vesicular Stomatitis Virus (VSV) system was used to generate SARS-CoV-2 pseudovirus (PV) as previously described [29]. Human embryonic kidney 293T (HEK293T) cells were cultured in Gibco Dulbecco’s Modified Eagle Medium (DMEM) supplemented with 10% FBS and 1% penicillin–streptomycin and maintained at 80–90% confluence during the experiment. The next day, the culture medium was replaced with Gibco Opti-MEM and incubated for 20 min. Opti-MEM was used before transfection to enhance transfection efficiency and minimize cytotoxicity [30].

Cells were transfected with SARS-CoV-2 spike plasmid (wild-type or N481K mutant) and incubated for 4 h, after which the transfection medium was replaced with DMEM containing 5% FBS. Cultures were maintained at 37 °C in a humidified 5% CO₂ incubator. After 24 h, cells were examined for the presence of syncytia, indicative of spike expression. Transfected cells were then infected with pseudotyped ΔG-luciferase (G*ΔG) VSV (Kerafast, Ref. EH1025-PM) at a multiplicity of infection of approximately 3–5. When most cells exhibited cytopathic effect (24–30 h post-infection), the pseudovirus-containing supernatant was harvested and clarified by centrifugation at 300×g for 10 min. Supernatants were aliquoted and stored at − 80 °C. For pseudovirus titration, HEK293T-ACE2 cells (BEI Resources) were seeded at 1 × 10⁶ cells/ml in complete DMEM. Serial dilutions of pseudovirus were prepared (50 µl diluted virus + 50 µl cell suspension per well) in a 96-well plate and incubated for 2 h. After incubation, 100 µl of complete DMEM was added, and cells were cultured for 48 h. Cells were then lysed with 30 µl of 1× cell lysis buffer (Promega), followed by the addition of 50 µl luciferase reagent (Promega). Luminescence was measured using a plate reader (Tecan Infinite 200 PRO), and viral titers were calculated based on relative light units (RLU).

For neutralization assays, heat-inactivated serum samples (50–200-fold dilutions) were prepared in 60 µl of DMEM and incubated with 100 µl of pseudovirus (1–2 × 10⁶ RLU/100 µl) for 30 min at room temperature. The final 160 µl mixture was distributed in triplicate wells of a 96-well culture plate. HEK293T-ACE2 cells were added at 1 × 10⁶ cells/ml and incubated for 48 h before luminescence was measured using the same plate reader and percentage inhibition was calculated for each sample.

#### Statistical analysis

Statistical analyses were conducted using GraphPad Prism version 7.05. Comparisons between wild-type and N481K were performed using paired tests. Data normality was assessed prior to analysis; accordingly, paired t-tests were applied for normally distributed data, while the non-parametric Wilcoxon matched-pairs signed-rank test was used when normality assumptions were not met. A p value < 0.05 was considered statistically significant.

## Results

### Temporal and geographic circulation of S: N481K (2020–2025)

To track the circulation of the N481K amino acid substitution in the SARS-CoV-2 spike protein, genomic data were retrieved from the CoV-Spectrum database [31]. This platform enables tracking of SARS-CoV-2 mutations and lineages by providing their prevalence, geographic distribution, and temporal trends. Data spanning January 6, 2020, to September 10, 2025, were analyzed.

#### Global distribution

**Fig. 1:**
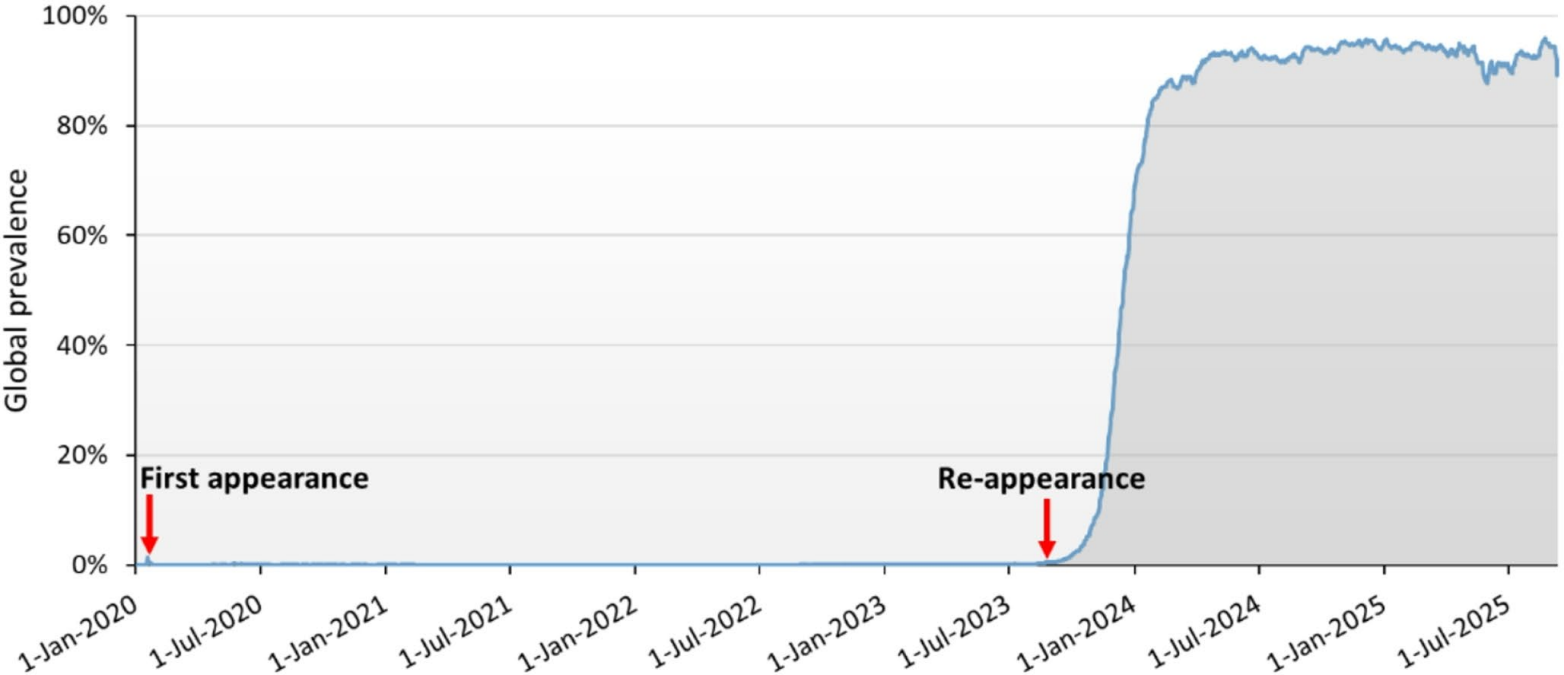
Temporal dynamics of the SARS-CoV-2 S: N481K mutation. The global detection of S: N481K occurred in early 2020. After remaining rare globally for several years, the mutation re-emerged in week 38 of 2023, with prevalence rising sharply and surpassing 60% by late 2023. The shaded area represents the proportion of sequenced samples carrying S: N481K over time

A total of 17,016,111 SARS-CoV-2 sequences deposited between January 2020 and September 2025 were analyzed, of which 703,777 (4.14%) carried the S: N481K mutation. The mutation was first detected in January 2020 in the United States and subsequently identified in Qatar on April 21, 2020, where it exceeded 50% prevalence by January 2021. Globally, the mutation remained rare (< 1%) but with a gradual upward trend. Starting from week 38 of 2023, its prevalence exceeded 1% and rose sharply, surpassing 60% by late 2023. By early 2024, the N481K mutation became dominant in the majority of circulating genomes, with its prevalence increasing from 71.5% in week one of 2024 to 94.4% in week 36 of 2025. By week 37 of 2025, a mild decline to ~ 87.5% was observed, marking the first notable decrease since its dominance (Fig. 1).

**Fig. 2:**
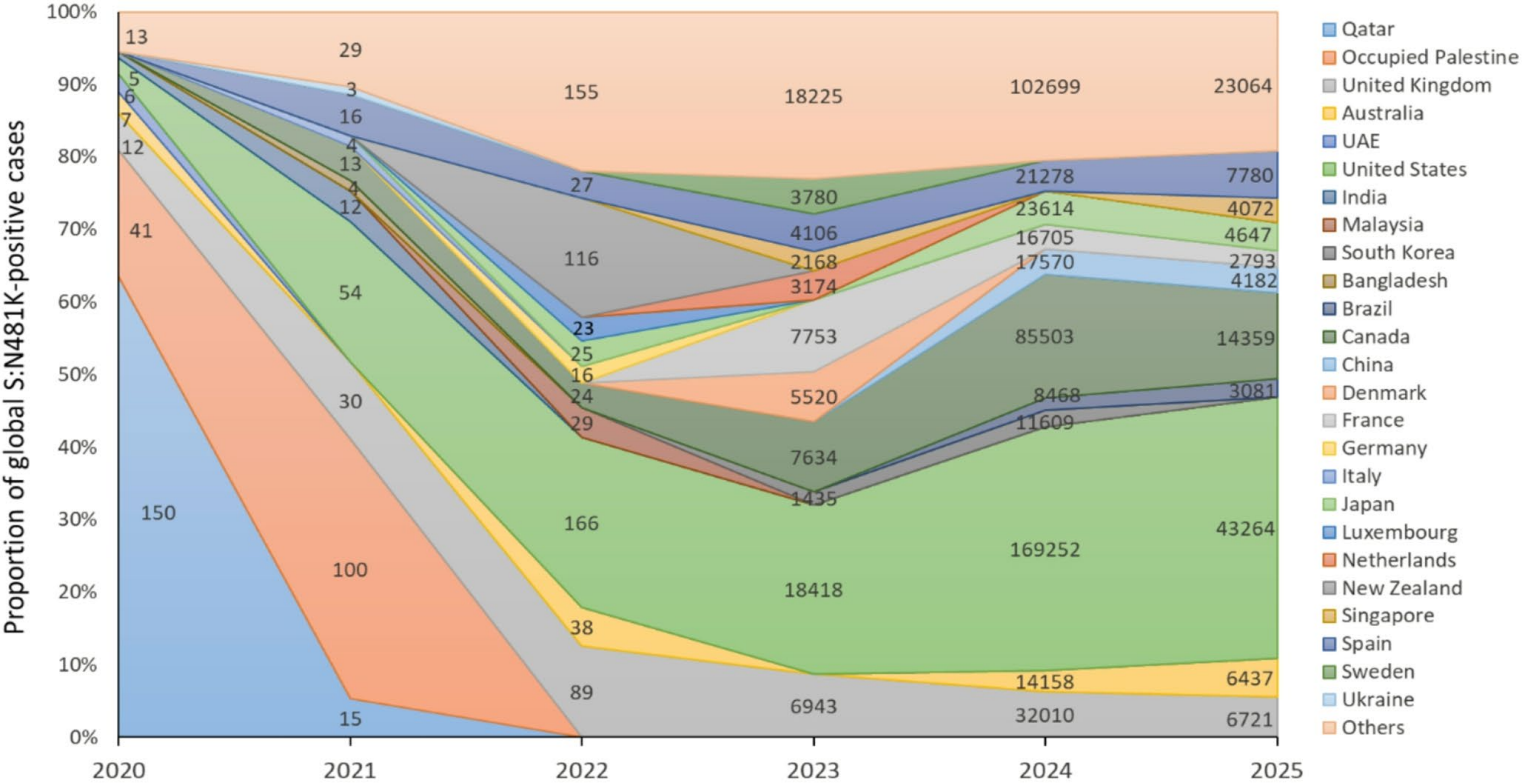
Geographic distribution of SARS-CoV-2 S:N481K mutation over time. Shown is the relative contribution of different countries to all globally sequenced SARS-CoV-2 genomes carrying the S:N481K mutation between 2020 and 2025. The y-axis represents the percentage of global S:N481K-positive sequences originating from each country. While Middle Eastern countries contributed disproportionately during the early pandemic phase, the distribution became more diverse after the global re-expansion of S:N481K in 2023

Geographically, the contribution of different countries to the global pool of S: N481K-positive sequences shifted substantially over time (Fig. 2). In the early pandemic phase (2020–2021), Qatar and Occupied Palestine accounted for the majority of detected cases, reflecting localized circulation in the Middle East. By 2022–2023, however, the United States emerged as a major contributor, alongside detections in Europe (United Kingdom, Germany, Italy) and Asia (India, Bangladesh, Malaysia, South Korea).

Across lineages, S:N481K has been most enriched in JN.1 (11.72%) and KP.3.1.1 (8.34%). Between January and September 2025, the mutation was identified in 93.23% (*n* = 120,420) of all sequenced samples. Lineages XEC (8.42%), LP.8.1.1 (5.93%), and NB.1.8.1 (5.54%) exhibited the highest overall prevalence of the mutation.

#### Circulation in Qatar

In Qatar, the S: N481K was first detected in April 21, 2020, and rapidly expanded with 150 cases reported by the end of 2020, representing 9.25% of global N481K cases. The B.1 lineage accounted for nearly all early detections (98%). In total, 440 sequences harboring S: N481K were reported in Qatar between 2020 and September 2025. The mutation circulated widely between April 2020 and January 2021, and then disappeared, only to re-emerge in late 2023. From March 2024 onward, all sequenced samples from Qatar carried the mutation.

In 2024, S: N481K was present in 100% of sequences (171/171), predominantly within lineages NT.1 (28.07%), MV.1 (5.85%), and LF.7.1 (5.26%), among others. In 2025 (as of September 10), all sequences continued to carry the mutation (100/100), with notable representation in XFG.7 (24.0%), XFG.5.1 (10.0%), NW.1 (7.0%), and XFG.6 (6.0%), among other lineages.

### Molecular docking and structural modeling of the S: N481K mutation

#### Structural analysis of the wild-type and N481K variant

**Fig. 3:**
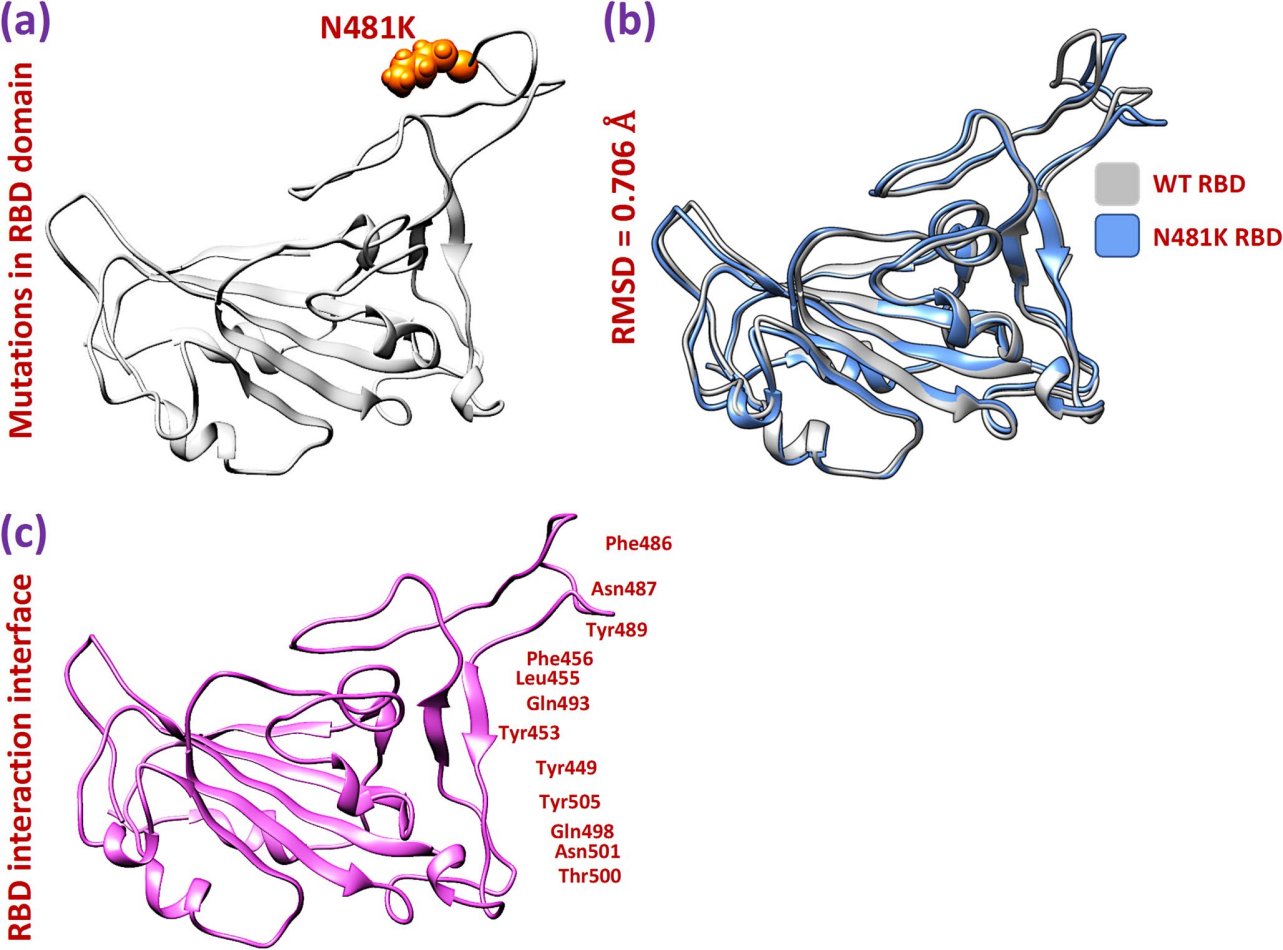
Modeling and structural variation analysis. (**a**) showing the position of the modeled mutation in RBD. (**b**) showing superimposed structures of wild-type and N481K RBD. (**c**) showing the binding interface residues of RBD

To understand the structural consequences behind the apparent adaptive advantage of the N481K substitution and its widespread prevalence, molecular modeling and docking analyses were conducted. Utilizing the chimera software, we modeled the S: N481K mutation in the RBD and compared it with the wild-type protein, recording differences in RMSD as shown in Fig. 3a. Our analysis revealed a notable structural deviation of 0.706 Å between the wild-type RBD and the N481K RBD (Fig. 3b). This structural alteration suggests a potential impact on the RBD’s conformation, which could influence its binding affinity with human ACE2. To delve deeper into the consequences of these mutations on the RBD-ACE2 interaction, we performed molecular docking analysis. Figure 3c illustrates the defined binding interface of the RBD for the docking process with human ACE2, providing valuable insights into the potential implications for viral infectivity.

#### Comparative bonding analysis between wild-type and N481K RBD-ACE2

**Fig. 4:**
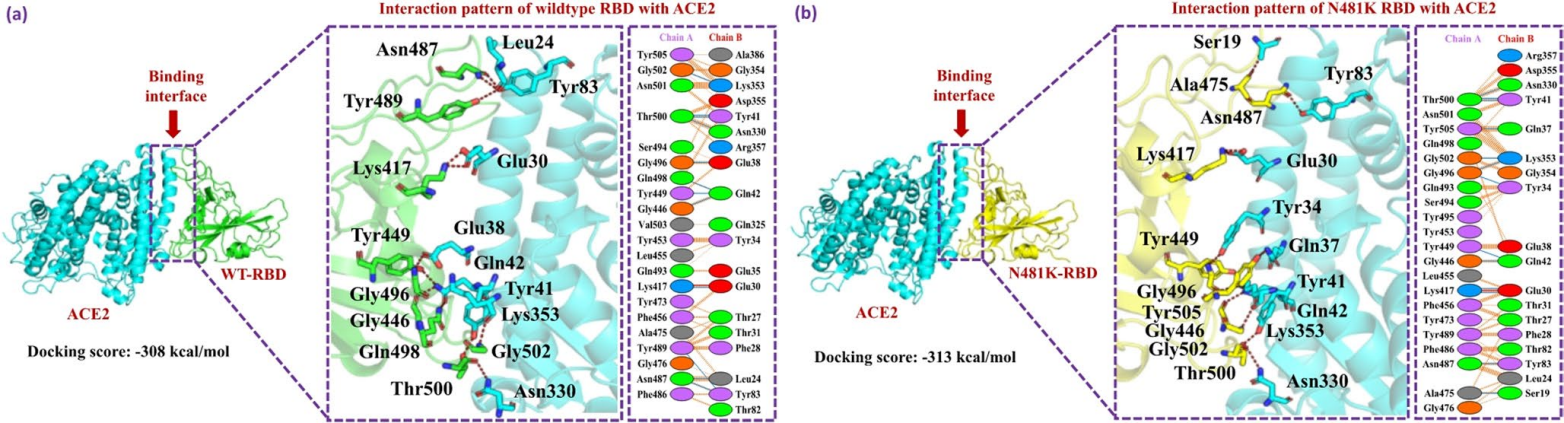
Comparison of wild-type and N481K-ACE2 bonding network. (**a**) Represents hydrogen bonds as sticks in wild-type RBD-ACE2 complex. (**b**) Represents hydrogen bonds as sticks in the N481K RBD-ACE2 complex

Further, we conducted a comparative analysis of the binding between the wild-type and mutant RBD proteins using the HDOCK server. The wild-type RBD-ACE2 complex exhibited a predicted score of -308 kcal/mol. Analysis of the binding interface using the PDBsum server uncovered the existence of 12 hydrogen bonds, 1 salt bridge, and 170 non-bonded contacts. The crucial amino acid residues involved in forming these hydrogen bonds included Gly502-Lys353, Thr500-Tyr41, Thr500-Asn330, Gly496-Glu38, Gln498-Gln42, Tyr449-Gln42, Gly446-Gln42, Lys417-Glu30, Tyr489-Tyr83, Asn487-Leu24, and Asn487-Tyr83. Additionally, a salt bridge was identified between Lys417 and Glu30 residues (Fig. 4a). In comparing the binding network of the mutant (N481K) RBD-ACE2 complex with the wild-type RBD-ACE2 complex, docking simulations were performed. The HDOCK server predicted a binding score of -313 kcal/mol for the mutant complex, indicative of a significant increase in docking scores and suggesting a heightened binding affinity compared to the wild-type. This observed trend aligns with findings reported in previous SARS-CoV-2 variants [32, 33]. Conducting an interface analysis through PDBsum, it was revealed that the N481K mutation notably enhanced the binding affinity of the RBD with the human ACE2 receptor characterized by the formation of 1 salt bridge, 13 hydrogen bonds, and 155 non-bonded contacts. Specifically, hydrogen bonds were observed between Thr500-Asn330, Thr500-Tyr41, Tyr505-Gln37, Gly502-Lys353, Gly496-Lys353, Gly496-Tyr34, Tyr449-Gln42, Gly446-Gln42, Lys417-Glu30, Asn487-Tyr83, and Ala475-Ser19 amino acid residues. Additionally, salt bridges were formed between Glu30 and Lys417 residues (Fig. 4b). These indicate a heightened affinity of the N481K RBD mutant for human ACE2 in comparison to the wild-type RBD.

#### Dynamic stability and compactness analysis of wild-type and mutant complexes

**Fig. 5:**
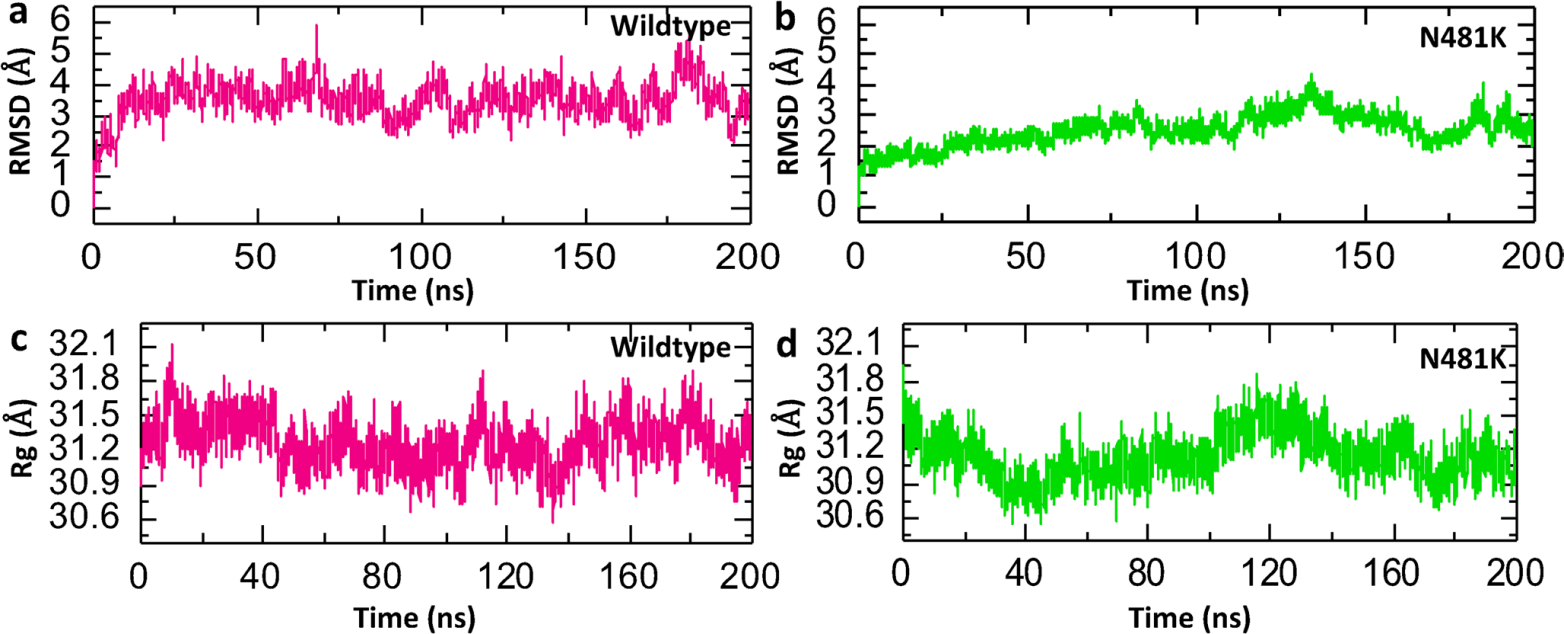
Dynamic stability and compactness analysis of the wild-type and N481K-RBD-ACE2 complexes. (**a**) Showing the RMSD value of wild-type complex. (**b**) Showing the RMSD value of N481K complex. (**c**) Showing the Rg value of wild-type complex (**d**) Showing the Rg value of N481K complex

To assess the structural stability of protein complexes, we conducted calculations for the Root Mean Square Deviation (RMSD) over time. A lower RMSD value signifies a more stable structure, whereas a higher RMSD value implies increased deviation and reduced stability. The results depicted in Fig. 3 demonstrate a more consistent behavior in the N481K complex when compared to its wild-type counterparts. The wild-type RBD-ACE2 complex exhibited stability at 5ns and maintained a steady state throughout the entire 200ns simulation, with noticeable fluctuations at various time points, resulting in an average RMSD value of approximately 4 Å (Fig. 5a). In contrast, the N481K RBD-ACE2 complex underwent initial equilibration at 2ns and remained stable throughout the entire simulation duration, displaying significant convergence compared to the wild-type complexes. The average RMSD value for the N481K complex was recorded to be 3 Å (Fig. 5b). The lower RMSD in the N481K complex, in comparison to the wild type, suggests a more stable confirmation of the mutant complex. Additionally, we assessed the structural compactness of both complexes by calculating the Radius of Gyration (Rg), which is a metric for a protein’s folded state compactness, providing insights into its overall shape and structure during folding [34]. Interestingly, both the wild-type and mutant complexes exhibited a similar Rg pattern. Notably, the wild-type complex displayed a slightly higher Rg value at 31.2 Å, in contrast to the N481K complex, which had a value of 31.0 Å (Fig. 5d, c). These findings align with the docking results, reinforcing the notion of the N481K RBD’s enhanced affinity for ACE2 compared to its wild-type counterpart.

#### Residual fluctuation and hydrogen bonding analysis of wild-type and mutant complexes

**Fig. 6:**
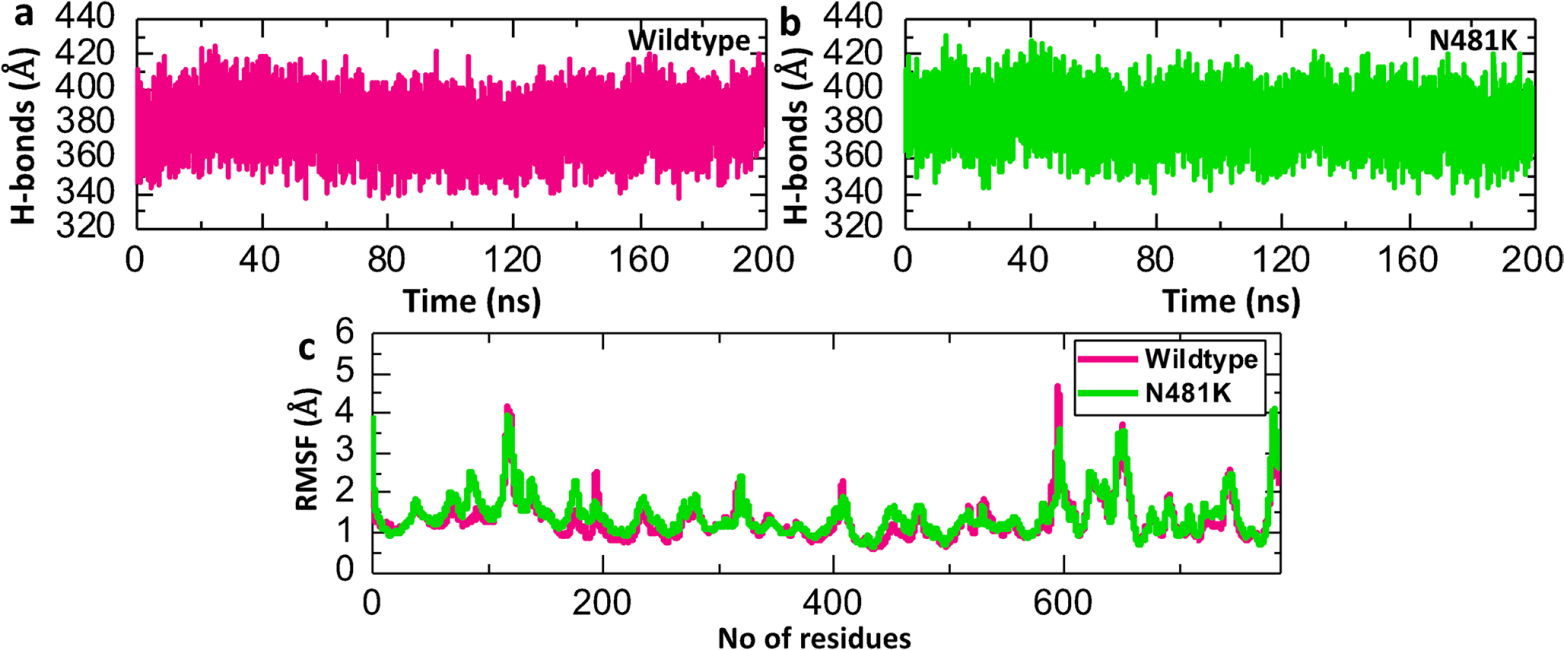
Residual fluctuation and hydrogen bonding analysis of wild-type and N481K-RBD-ACE2 complexes. (**a**) Showing the average hydrogen bonds between the wild-type RBD and ACE2. (**b**) Showing the average hydrogen bonds between the N481K RBD and ACE2. (**c**) Showing the RMSF value of both wild-type and mutant complexes

Hydrogen bonding and residue flexibility play critical roles in protein–protein interaction stability. We assessed the strength of both the wild-type and mutant complexes by conducting an average hydrogen bonds analysis. Figure 6 illustrates that both the wild-type and mutant complexes exhibit a comparable hydrogen bonding pattern, with an average of 380 hydrogen bonds (Fig. 6a, b). Moreover, we employed the calculation of RMSF of backbone C-alpha atoms to gain insights into the flexibility of different regions within a protein structure. Although both complexes share a similar RMSF pattern, it is noteworthy that the average RMSF value of the N481K complex is lower than that of the wild-type complex (Fig. 6c).

#### Binding free energy calculation

To overcome the limitations associated with traditional alchemical methods, current research adopts the MM/PBSA approach, known for its cost-effectiveness and reduced computational demands. In this study, we used the MM/PBSA approach to compute the binding free energy of wild-type and mutant RBD-ACE2 complexes based on a 200ns simulation trajectory. Table 1 displays the Van der Waals and electrostatic energies for the wild-type RBD-ACE2 complex as -76.85 ± 0.67 kcal/mol and − 577.02 ± 5.29 kcal/mol, respectively. In contrast, the N481K complex showed energies of -91.17 ± 0.78 kcal/mol and − 804.61 ± 4.64 kcal/mol. Additionally, the total binding free energy for the wild-type RBD-ACE2 complex was − 43.09 ± 0.98 kcal/mol, whereas for the N481K RBD-ACE2 complex, it was − 77.07 ± 0.90 kcal/mol. These results indicate a significantly lower binding free energy for the mutant complex, suggesting a higher affinity between RBD and human ACE2.

**Table 1:** Binding free energy of wild-type and N481K complexes

MM/PBSA		
Parameters	Wild-type RBD-ACE2	N481K RBD-ACE2
ΔEvdw	-76.85 ± 0.67	-91.17 ± 0.78
ΔEele	-577.02 ± 5.29	-804.61 ± 4.64
EPB	620.04 ± 4.98	828.94 ± 4.17
ENPOLAR	-9.26 ± 0.05	-10.23 ± 0.03
Delta G Gas	-653.87 ± 5.58	-895.78 ± 4.57
Delta G Solv	610.77 ± 4.95	818.70 ± 4.16
∆G total	-43.09 ± 0.98	-77.07 ± 0.90

### Functional assessment of the neutralization activity against the N481K mutant PV

#### Demographics of the study groups

A total of 137 samples were included in the study, of which, 48 samples were obtained from SARS-CoV-2–infected patients, 16 from individuals vaccinated with mRNA-based vaccines [mRNA-1273 (Moderna) and/or BNT162b2 (Pfizer–BioNTech)], 45 from individuals vaccinated with the inactivated virus vaccine [BBIBP-CorV (Sinopharm)], and 28 from recipients of the viral vector vaccine [ChAdOx1 nCoV-19 (AstraZeneca)]. Table 2 summarizes the studied population categories.

**Table 2:** Description of the study participants

Group	Samples no.	Age median (IQR)	Gender no. (%)
SARS-CoV-2 infected patients	48	46 (35–55)	M: 42 (87.5) F: 6 (12.5)
Vaccinated individuals			
mRNA [mRNA-1273 (Moderna)/BNT162b2 (Pfizer–BioNTech)]	16	NA	M: 15 (94.8) F: 1 (6.3)
Inactivated virus vaccine [BBIBP-CorV (Sinopharm)]	45	NA	NA
Viral vector vaccine [ChAdOx1 nCoV-19 (AstraZeneca)]	28	NA	NA
Total no. of samples	137		

#### Overall neutralization against the WT-S and N481K-S pseudoviruses

**Fig. 7:**
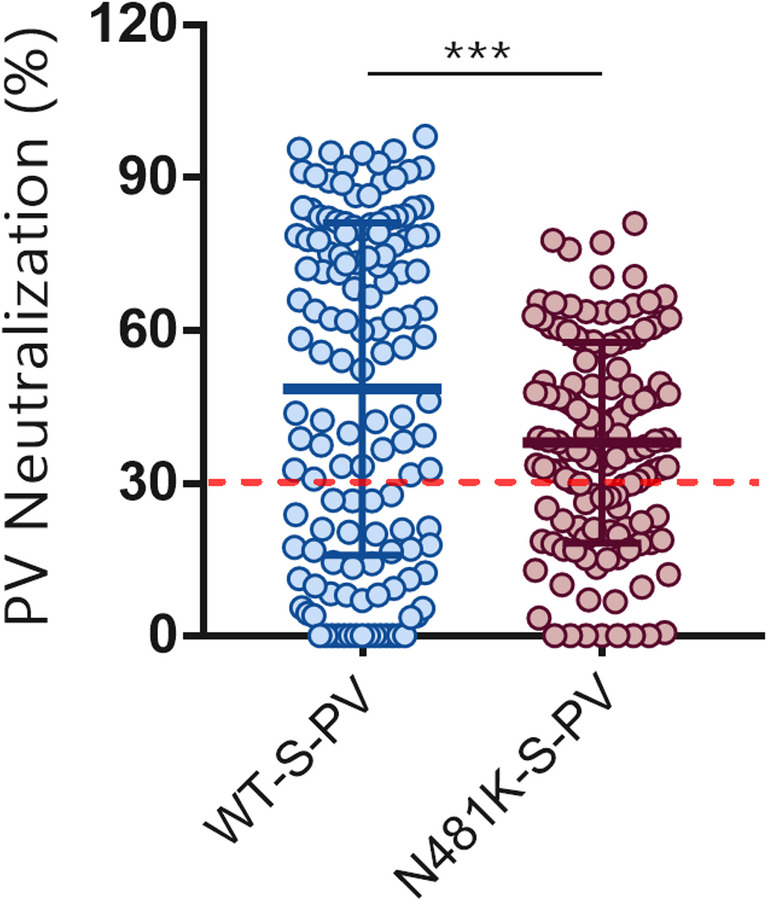
Neutralization of WT-S-PV and N481K-S-PV in the overall study population. Serum from all participants (*n* = 137) was tested using pseudovirus neutralization assay against pseudoviruses carrying either the wild-type SARS-CoV-2 spike (WT-S-PV) or the N481K mutant spike (N481K-S-PV). The Wilcoxon matched-pairs signed-rank test showed a significant decrease in neutralization against the N481K mutant (median difference = − 14.47; ***p = 0.0002, two-tailed) compared to the WT-S-PV. Each dot represents an individual sample; horizontal red lines indicate median values, and the dashed red line indicates the 30% neutralization threshold

To assess the overall neutralizing activity of the collected sera (*n* = 137), we compared the neutralization activity against pseudoviruses carrying the wild-type spike (WT-S-PV) with those harboring the N481K mutation (N481K-S-PV) **(**Fig. 7**)**. The average neutralization against WT-S-PV was 48.5% (95% CI: 42.98–54.02), while neutralization against the N481K-S-PV was reduced to 37.9% (95% CI: 34.6–41.25). The distribution of responses was broad for both PVs, with values ranging from 0% to 98.14% for WT-S-PV and from 0% to 81% for N481K-S-PV. The median neutralization against WT-S-PV was 55.7%, compared to 38.28% for N481K-S-PV, indicating a significant downward shift in neutralization potency. Likewise, the IQR highlighted reduced neutralization in the mutant group (WT-S -PV: 16.66–78.87% vs. N481K-S-PV: 22.42–51.09%). Among samples showing positive neutralization to the WT-S-PV (≥ 30% PV inhibition, *n* = 89), the decline in the mean PV neutralization was more evident. It significantly decreased from 69.76 (95% CI: 65.86–73.67) for wild-type (WT-S-PV) to 42.47% (95% CI: 38.58–46.37) for N481K mutant (N481K-S-PV) (*p* < 0.0001).

#### Neutralization responses across infected and vaccinated groups

**Fig. 8:**
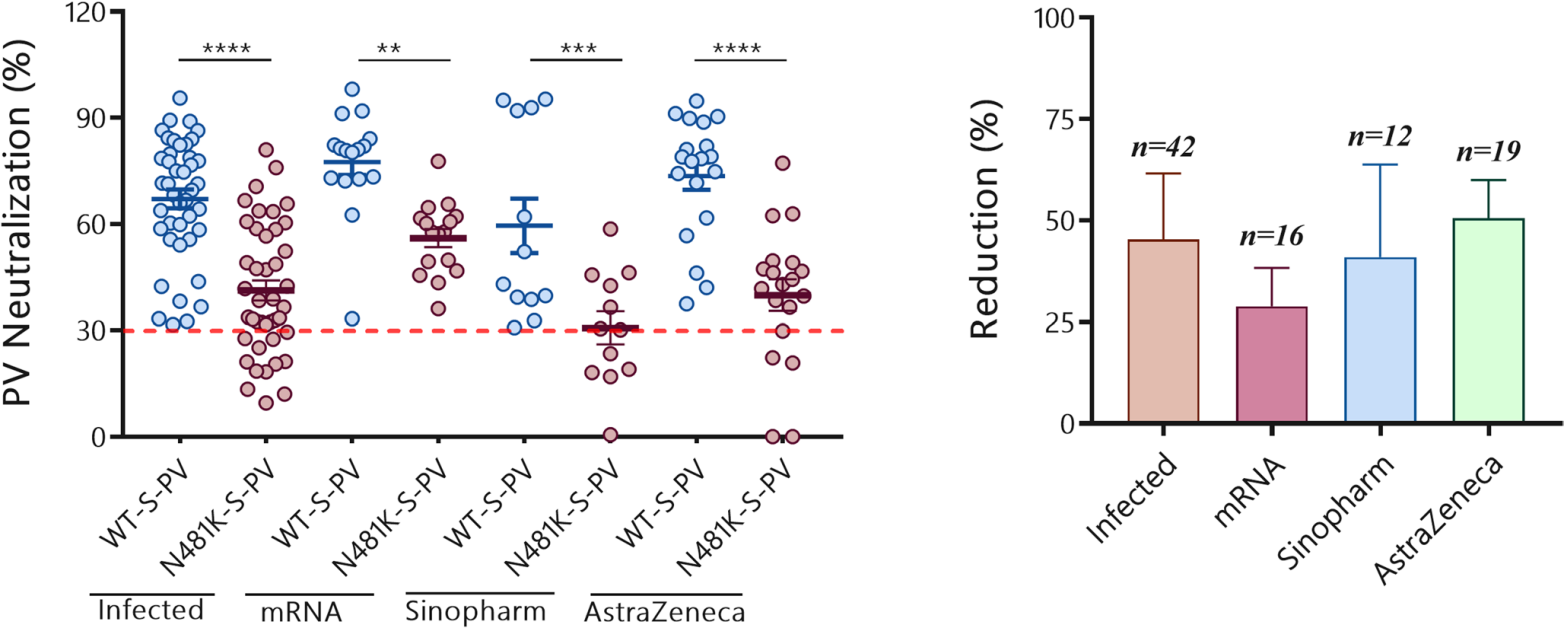
Neutralization of WT-S-PV and N481K-S-PV across infected and vaccinated cohorts.** (a)** Neutralization activity of sera from SARS-CoV-2–infected individuals (*n* = 42) and recipients of mRNA (*n* = 16), Sinopharm (*n* = 12), or AstraZeneca (*n* = 19) vaccines against pseudoviruses carrying the wild-type spike (WT-S-PV) or the N481K mutant (N481K-S-PV). Only samples exhibiting positive neutralization against WT-S-PV (≥30% inhibition) were included in this analysis. Each dot represents an individual sample; horizontal bars indicate group means ± SEM. The dashed red line denotes the 30% cutoff used to define positive neutralization. Only samples with WT-S-PV neutralization > = 30 are plotted. ***p < 0.001. **(b)** Percent reduction in neutralization against N481K-S-PV relative to WT-S-PV in the same cohorts. Bars represent means ± SEM, with sample sizes indicated above

Among samples showing positive neutralization to the WT-S-PV (*n* = 89), we compared responses across convalescent individuals and those vaccinated with mRNA, inactivated virus (Sinopharm), or viral vector (AstraZeneca) vaccines **(**Fig. 8a**)**. In the infected group (*n* = 42), neutralization of WT-S-PV was strong (mean 67.1%, 95% CI: 61.8–72.4), but dropped substantially against the N481K-S-PV (mean 41.2%, 95% CI: 35.5–47.0). A similar pattern was observed in the mRNA group (*n* = 16), however, with higher retention of neutralization activity against the N481K mutant compared to other cohorts. Neutralization in mRNA-vaccinated group decreased from a mean of 77.5% (95% CI: 69.7–85.3) for WT-S-PV to 56.0% (95% CI: 50.6–61.5) for N481K-S-PV. On the other hand, the Sinopharm group (*n* = 12) showed weaker and more variable responses overall (mean 59.5% vs. 30.7%), while the AstraZeneca group (*n* = 19) displayed intermediate levels (73.5% vs. 39.9%). Across all cohorts, neutralization of the N481K-S-PV was consistently and significantly reduced relative to WT-S-PV, underscoring the immune evasive potential of this mutation.

When expressed as percent reduction **(**Fig. 8b**)**, distinct patterns emerged. The mean reduction in neutralization was lowest among mRNA-vaccinated individuals (mean 28.7%, 95% CI: 21.0–36.5), indicating partial preservation of cross-neutralizing activity. In contrast, greater reductions were observed in convalescent individuals (mean 40.7%, 95% CI: 32.6–48.8) and Sinopharm recipients (mean 43.8%, 95% CI: 26.0–61.6), while the AstraZeneca group showed the largest average reduction (mean 48.0%, 95% CI: 35.2–60.8).

To contextualize these reductions, we assessed the proportion of sera exceeding defined thresholds. Against WT-S-PV, high neutralization (> 70%) was most frequent in the mRNA vaccinated group (88%), followed by AstraZeneca vaccinated (50%), infected (44%), and Sinopharm vaccinated (9%) groups. Using a > 50% cutoff, responses were retained in 94% of mRNA, 73% of infected, 57% of AstraZeneca, and 13% of Sinopharm samples. In contrast, against the N481K-S-PV, high neutralization (> 70%) was rare across all cohorts (≤ 8%). At the 50% cutoff, a substantial fraction of mRNA sera (62.5%) retained activity, compared to 33% of infected, 14% of AstraZeneca, and 9% of Sinopharm samples. At the more permissive 30% cutoff, all mRNA sera (100%) retained activity, whereas infected (88%), AstraZeneca (68%), and Sinopharm (44%) showed lower retention. Overall, mRNA vaccination generated the most potent responses against WT-S-PV and showed the highest proportion of sera retaining ≥ 50% activity against the N481K mutant, although the mutation still markedly reduced neutralization across all groups.

## Discussion

In this study, we investigated the circulation and impact of the SARS-CoV-2 spike mutation N481K by integrating computational methods and functional assays. Mutations in the RBD of the spike protein have been of a particular interest due to their influence on the virus’s affinity to ACE2 receptor and impact on susceptibility to neutralizing antibodies. Several amino acid changes within the RBD, including K417N/T, E484K, N501Y, L452R, T478K, and Q498R, have been reported to alter receptor engagement and antibody recognition, weakening neutralization while preserving or enhancing ACE2 binding through compensatory effects [3]. Investigating additional, less-characterized mutations such as N481K is therefore essential to understand their contribution to viral evolution and transmissibility.

Tracking data from global SARS-CoV-2 sequenced genomes revealed an early emergence of the N481K mutation locally. Qatar, specifically, represented one of the earliest hotspots of N481K circulation (2020–2021), where it was detected in the backbone of B.1.428 and B.1 lineages [13]. Globally, this mutation remained sporadic until mid-2022, indicating limited transmission or fitness. However, a sharp expansion was detected from mid-2023, with a ratio surpassing 60% of all sequenced samples. The N481K mutation became nearly fixed in globally dominant variants, maintaining a prevalence of 71–94% until mid-2025. Geographically, early circulation was concentrated in the Middle East, particularly Qatar and Occupied Palestine, before global dissemination to the United States, Europe, and Asia. This sustained global rise may suggest strong positive selection pressure favoring the N481K substitution, likely reflecting a fitness advantage that could have enabled its near-fixation in globally dominant Omicron-descendent lineages.

Structural modeling and molecular docking of N481K mutation further supported these observations. We found a measurable conformational deviation (0.706 Å RMSD) between the wild-type and N481K RBD, suggesting a potential alteration in conformation thereby influencing its interaction with the human ACE2 receptor. Additionally, the N481K–ACE2 complex had a more favorable binding score than the wild-type complex, suggesting enhanced receptor affinity. Molecular dynamics simulations revealed improved stability and compactness of the N481K complex, reflected by lower RMSD and the slightly reduced Rg. Moreover, the average RMSF value of the N481K complex was marginally lower than that of the wild-type complex, further implying decreased flexibility, contributing to a more rigid and stable interface with ACE2. Importantly, the significantly lower binding free energy of the N481K complex reflects a stronger interaction with human ACE2, which could correlate with enhanced viral infectivity. Collectively, these findings highlight the potential functional consequences of the N481K mutation on spike-ACE2 interactions and provide insight into its possible role in increased transmissibility. Many SARS-CoV-2 RBD mutations were found to reshape the conformational and energetic landscape of receptor binding through a combination of local structural adjustments and long-range epistatic effects [3]. Importantly, understanding the functional effect of an individual mutation cannot be understood apart from the broader structural and energetic network of other RBD mutations. For instance, although K417N/T, E484K, and S375F mutations reduce receptor binding to the ACE2, the virus frequently acquires compensatory mutations that restore or even increase ACE2 affinity, such as the Q498R+N501Y double mutations [35]. From the epidemiological and computational data, the N481K mutation is suggested to exert a synergic effect with other high-affinity RBD substitutions, rather than compensating effect.

To complement the computational analysis, and assess whether the N481K has a dual function via enhancing spike–ACE2 binding alongside mediating immune evasion, we evaluated its impact on antibody-mediated neutralization using sera from SARS-CoV-2-infected and vaccinated individuals. Consistent with the molecular modeling, we found that the neutralization against wild-type PV was notably higher (average 69.76%) than against the N481K mutant PV (average 42.47%) in all groups. This indicates that this mutation could compromise antibody recognition and reduces viral entry inhibition.

Analysis of neutralization per group showed that mRNA-vaccinated individuals displayed the strongest neutralizing responses against the wild-type PV and maintained the highest neutralization against the N481K mutant PV as well. This was followed by AstraZeneca-vaccinated then SARS-CoV-2 infected individuals. Despite the significant decline in neutralization against the mutant PV across all groups, the mRNA-vaccinated cohort retained the highest level of neutralizing potency. This could indicate a partial preservation of epitope recognition possibly owing to the broad antibody repertoire elicited by mRNA vaccines [36]. This observation is consistent with other reports from our groups and others, where mRNA vaccines (BNT162b2, mRNA-1273) contributed to the highest and most durable neutralization against the WT strain and VOCs whereas vector-based and inactivated-virus vaccines induce lower titers and greater fold-reduction [37–41]. Likewise, convalescent sera alone exhibited markedly reduced neutralization against immune-evasive variants such as Beta, Delta, and Omicron [42].

Although the N481K alone may not completely abrogate antibody binding, it contributes to measurable immune evasion, particularly in non-mRNA-induced immunity supporting its adaptive significance in viral persistence and spread. In fact, this immune escape accompanied with enhanced affinity is a hallmark of SARS-CoV-2 adaptive evolution, and has been observed for other mutations such as E484K, Q498R+N501Y, and other VOC-defining RBD substitutions [3, 43]. In addition, several studies proposed that increased rigidity within the receptor-binding motif (RBM) enhances receptor interaction while concurrently reducing antibody accessibility and therefore, compromising optimal antibody recognition [44, 45]. Taken together, our results suggest that N481K could play a role in virus adaptation, enabling SARS-CoV-2 to maintain strong receptor engagement while escaping humoral immune responses at the same time.

## Conclusion

In conclusion, this study provides integrative evidence that the N481K mutation is associated with increased predicted spike–ACE2 binding affinity while reducing antibody-mediated neutralization, suggesting a potential synergistic effect on SARS-CoV-2 viral fitness and persistence. The structural, functional, and epidemiological data suggest an adaptive contribution of N481K to the ongoing evolution of SARS-CoV-2. However, our findings are limited by the in silico modeling and small sample-sized neutralization datasets, and thus, might not capture the full spectrum of immune interactions. Indeed, combining high-resolution structural analyses with variant co-mutation modeling is warranted to better understand the impact of N481K with other mutation combinations in RBD as well as non-RBD, all of which contribute to viral evolution and affect binding and immune escape.

## Supplementary Information

Below is the link to the electronic supplementary material.

Supplementary Material 1 (DOCX 12.0 KB)

## Data Availability

All data supporting the findings of this study are available within the paper.

## Declarations

**Declaration of using AI-assisted technologies**

During the preparation of this manuscript, the author used ChatGPT (OpenAI) to improve the structure, readability, and overall flow of the text. After using this tool, the author carefully reviewed AI-generated text, and edited the content as needed. The authors take full responsibility for the content of the published article.
