# Supplementary material for: Functional and structural characterization of the SARS-CoV-2 spike N481K mutation
Source: Arch Virol. 2026 Jun 4;171(7):198. doi: 10.1007/s00705-026-06652-y (PMC13236748; doi:10.1007/s00705-026-06652-y)
Supplement: Supplementary file 1 — Supplementary Material 1 (DOCX 12.0 KB) [file 705_2026_6652_MOESM1_ESM.docx]

**Supplementary information**

MMGBSA.py script

| Per-residue GB and PB decomposition  &general  startframe=5000, endframe=10000, interval=10,  /  &gb  igb=5, saltcon=0.150,  /  &pb  inp=1, istrng=0.15, radiopt=0,  / |
| --- |
